# The Role of Arch Compression and Metatarsophalangeal Joint Dynamics in Modulating Plantar Fascia Strain in Running

**DOI:** 10.1371/journal.pone.0152602

**Published:** 2016-04-07

**Authors:** Kirsty A. McDonald, Sarah M. Stearne, Jacqueline A. Alderson, Ian North, Neville J. Pires, Jonas Rubenson

**Affiliations:** 1 School of Sport Science Exercise and Health, The University of Western Australia, Crawley, Perth, Western Australia, Australia; 2 Willetton Podiatry, Willetton, Perth, Western Australia, Australia; 3 Biomechanics Laboratory, Department of Kinesiology, The Pennsylvania State University, University Park, State College, Pennsylvania, United States of America; University of Zaragoza, SPAIN

## Abstract

Elastic energy returned from passive-elastic structures of the lower limb is fundamental in lowering the mechanical demand on muscles during running. The purpose of this study was to investigate the two length-modulating mechanisms of the plantar fascia, namely medial longitudinal arch compression and metatarsophalangeal joint (MPJ) excursion, and to determine how these mechanisms modulate strain, and thus elastic energy storage/return of the plantar fascia during running. Eighteen runners (9 forefoot and 9 rearfoot strike) performed three treadmill running trials; unrestricted shod, shod with restricted arch compression (via an orthotic-style insert), and barefoot. Three-dimensional motion capture and ground reaction force data were used to calculate lower limb kinematics and kinetics including MPJ angles, moments, powers and work. Estimates of plantar fascia strain due to arch compression and MPJ excursion were derived using a geometric model of the arch and a subject-specific musculoskeletal model of the plantar fascia, respectively. The plantar fascia exhibited a typical elastic stretch-shortening cycle with the majority of strain generated via arch compression. This strategy was similar in fore- and rear-foot strike runners. Restricting arch compression, and hence the elastic-spring function of the arch, was not compensated for by an increase in MPJ-derived strain. In the second half of stance the plantar fascia was found to transfer energy between the MPJ (energy absorption) and the arch (energy production during recoil). This previously unreported energy transfer mechanism reduces the strain required by the plantar fascia in generating useful positive mechanical work at the arch during running.

## Introduction

Running is an elastic gait, relying on the storage and release of elastic strain energy, primarily in tendons and ligaments, to reduce the mechanical demands on lower limb muscles [1, 2]. Arguably the most well-studied example in human locomotion is the Achilles tendon, which has been estimated to contribute as much as 35% of the mechanical energy required in running [3, 4]. Other elastic structures that function to reduce the cost of transport are the ligaments of the foot’s medial longitudinal arch (MLA). These include the plantar fascia (PLF), the long and short plantar ligaments, and the calcaneonavicular ligament. In a normative population, compressive loads are applied to the foot during the first half of stance phase, displacing the apex of the arch (typically depicted by the navicular bone) downwards, resulting in an increased displacement between the calcaneus and metatarsal heads. A passive stretch is subsequently experienced by the MLA ligaments and thus elastic energy is stored. When the arch recoils in the second half of stance, this elastic energy is returned. Recoil of the arch assists in generating the gravitation potential energy and kinetic energy required to accelerate the body’s center of mass. From cadaveric experimentation, Ker, Bennett [3] estimated that the combined elastic energy returned by MLA ligaments provides approximately 17% of the total mechanical energy expended when running at 4.5ms^-1^ and Stearne and colleagues [5] estimated that these structures provide approximately 8% of the mechanical energy required to run at 2.7ms^-1^. Additionally, Stearne, McDonald [5] showed that limiting the arch spring by the use of insoles that restrict arch compression results in an increase in metabolic energy cost proportional to the lost elastic work.

Although the spring function of the MLA has been proposed, a detailed understanding of its elastic mechanisms and the role of the individual MLA ligaments is lacking. Whilst several studies have acknowledged the elastic energy storage potential of the PLF [3, 6, 7], this ligament is primarily regarded for its role in providing integrity to the bony arch structure [8–13], and in supplying the rigidity required for the foot to function as a lever during propulsion [14–17]. The importance of the PLF in providing elastic energy storage and return may have been overlooked due to a lack of biomechanical analyses regarding its function in running. Such an understanding may have importance to clinicians prescribing treatment for conditions related to the foot (e.g. plantar fasciitis) or podiatrists prescribing foot orthotic interventions.

With an origin at the calcaneal tuberosity and insertions at the base of each proximal phalanx, the PLF is unique in that it can be stretched via both arch compression and metatarsophalangeal joint (MPJ) flexion (dorsiflexion, [18]). This feature of the PLF may allow it to function as an effective spring, as well as to modulate the elastic function of the foot under varying loading environments that impact upon arch compression and thus energy storage in other MLA ligaments. Furthermore, whether the PLF exhibits a similar spring function across different running foot strike techniques has not yet been investigated, however it has previously been proposed that elastic energy storage potential is greater in the MLA ligaments of forefoot strike (FFS) as opposed to rearfoot strike (RFS) runners [19].

Our aim was therefore to investigate how arch compression and MPJ dynamics affect the potential for the PLF to store and return elastic energy in running. Firstly, we sought to explore the spring function of the PLF and the relative importance of its two length-modulating mechanisms. This was achieved by estimating PLF strain as a function of arch compression and MPJ angle independently. Secondly, to better understand the importance of PLF strain to elastic energy storage and return, we assessed how MPJ-modulated strain responds when arch compression, and thus MLA elastic energy storage, is compromised (experimentally using custom made arch inserts). Finally, we assessed the above objectives in both FFS and RFS runners.

We hypothesized that the PLF would undergo length changes during the stance phase of running commensurate with a stretch-shorten spring function. When arch compression was compromised, we predicted that a higher maximum MPJ flexion angle and an earlier onset of MPJ extension at push off would occur; (a) to increase MPJ-induced PLF strain (compensating for lost strain across the arch), and (b) to generate greater positive MPJ power (compensating for lost positive mechanical work of the MLA). Furthermore, we hypothesized that FFS runners would exhibit greater peak PLF strain than RFS runners owing to their purported greater MLA elastic energy storage potential [19].

## Methods

### Participants

Eighteen healthy male distance runners participated in this study. Participants provided informed written consent and all procedures undertaken in this study were approved by the University of Western Australia (UWA) Human Research Ethics Committee prior to the commencement of testing (Approval ID: RA/4/1/4541). Nine subjects were habitual FFS runners (age 27.6±3.9 years; height 181.5±5.4cm; body mass 74.3±6.1kg) and nine were habitual RFS runners (age 26.1±4.2 years; height 183.7±6.8cm; body mass 78.6±7.3kg). Habitual foot strike technique was determined in accordance with the literature definition [20, 21].

Subjects were selected on the basis that they ran more than 30km per week. They were required to have sustained no serious lower limb injuries in the previous six months or have any pre-existing gait abnormalities. It was also required that participants not wear orthotics on a regular basis.

Prior to the testing session, participants met with a certified podiatrist who assessed the posture and morphology of both their left and right foot. Only participants considered to have neutral feet, as determined by the Foot Posture Index, which has been tested for both validity [22] and reliability [23], were included in the study.

### Joint Kinematics and Kinetics

**Fig 1:**
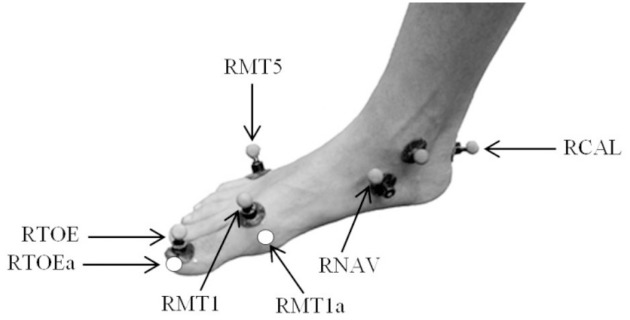
Modified foot marker set.

New Balance Minimus Zero Road shoes (mass ~180 grams; New Balance Athletic Shoe, Inc, Boston, USA) were used as standardized footwear and all subjects were within the shoe size range of US10-12. This model of shoe was selected because it minimally restricts MPJ flexion/extension angle and has a zero heel-to-toe drop, thus mass is not concentrated in a way that favors a FFS or RFS posture at initial ground contact. A series of retro-reflective markers were placed on the subject’s trunk, lower limbs and feet according to the marker set described by Besier, Sturnieks [24]. Two additional markers were combined with the foot marker set [24] (right foot only); one placed on the distal phalanx of the hallux (RTOE), and the other placed on the navicular tuberosity (RNAV; Fig 1). The testing shoe upper was modified to allow maker visibility, and for custom foot markers to be secured directly onto the foot rather than the shoe. The foot markers consisted of reflective spheres connected via magnets to their base. All bases were affixed to the relevant anatomical landmark of the foot, thus ensuring landmark positions remained consistent across all conditions.

An initial series of static and functional motion capture trials were used to define the subject’s anatomical coordinate system (10 camera Vicon Mx T40S, 250 Hz; Oxford Metrics, Oxford, UK). Left and right foot abduction/adduction and eversion/inversion angles were measured in order to construct the anatomical coordinate system of the rearfoot (posterior to the phalanges) as per Besier, Sturnieks [24]. The ankle joint center was defined as the midpoint between the medial and lateral malleoli. Functional trials were used to determine knee and hip joint centers and axes of rotation using a helical axis and sphere-fit, respectively. The MPJ center was defined as the midpoint of two virtual markers expressed relative to the anatomical foot segment (see below); the medial border of the first metatarsal head (RMP1a; Fig 1) and the lateral border of the fifth metatarsal head (RMP5a).

All participants completed a five minute warm up run, followed by three randomly ordered conditions; 1) shod running in standardized shoes, 2) insert running with custom inserts secured inside the standardized shoes, and 3) barefoot running. All conditions were completed on a force plate instrumented split belt treadmill (2000Hz; Bertec Corporation, Columbus, USA) at a speed of at 2.7 ms^-1^. This running speed was selected after pilot testing because it allowed subjects to run with minimal discomfort when using the insert, and did not incur high enough loads to mitigate the arch compression restricting effect of the insert. The chosen running speed also exceeds treadmill walk-to-run transition speeds, which are reported to occur around 2.0ms^-1^ [25, 26]. Subjects ran at a constant speed for five minutes in each condition (with the exception of barefoot running which was performed for approximately one minute to avoid discomfort), during which time data from five consecutive strides was recorded.

Three-dimensional (3D) lower limb kinematic and kinetic data were processed using Vicon Nexus software (Oxford Metrics, Oxford, UK) in accordance with Besier et al. [24]. Marker data were low-pass filtered at a cut-off frequency of 14Hz with a zero-lag forth-order Butterworth filter. This value was determined from a residual analysis. The same cut-off frequency was used to filter the Fy, Fz, Mx and My force plate channels to avoid artifacts in joint moments and powers [27]. However, due to a greater presence of lower frequency noise associated with the Fx and Mz channels, typical of treadbelt vibration in force plate instrumented treadmills, a 5Hz cut-off frequency was required. This filtering procedure was consistent across all participants and trials.

### Forefoot Model

In order to calculate MPJ kinematics and kinetics, a new forefoot model was developed. We defined the forefoot segment as the phalanges of the foot (anterior to the MPJ). Using a six-marker pointer wand, the medial border of the first metatarsal head (RMP1a; Fig 1) and the lateral border of the fifth metatarsal head (RMP5a) on the right foot were identified for each subject, and expressed relative to the anatomical rearfoot segment [24]. The mid-point between these virtual markers was used to determine the MPJ center (MPC). The same pointer wand was also used to define the location of the midpoint of the distal hallux (RTOEa; Fig 1) in order for it to be reconstructed in dynamic trials relative to a technical coordinate system defined by RMP1a, RMP5a, MPC and the hallux marker.

The forefoot segment was created in Vicon BodyBuilder software (Oxford Metrics, Oxford, UK), incorporating RMP1a, RMP5a, MPC, RTOEa. This segment allowed MPJ angles, net moments and powers in the sagittal plane to be determined for the right foot, based on the kinematics of the aforementioned physical and virtual landmarks. When the GRF center of pressure was anterior to the MPC, our model ascribed the GRF to the forefoot segment in the inverse dynamic calculations. Alternatively, when the center of pressure was posterior to the MPC, our model ascribed the GRF to the rearfoot segment. Inverse dynamic calculations assumed that the moments generated by the mass and inertia of the segment were negligible as per Stefanyshyn and Nigg [28]. A sensitivity analysis showed that this assumption is valid. Joint work at the MPJ was calculated by integrating the joint power curve with respective to time. MPJ work and power data were normalized to body mass.

### Plantar Fascia Strain Model

**Fig 2:**
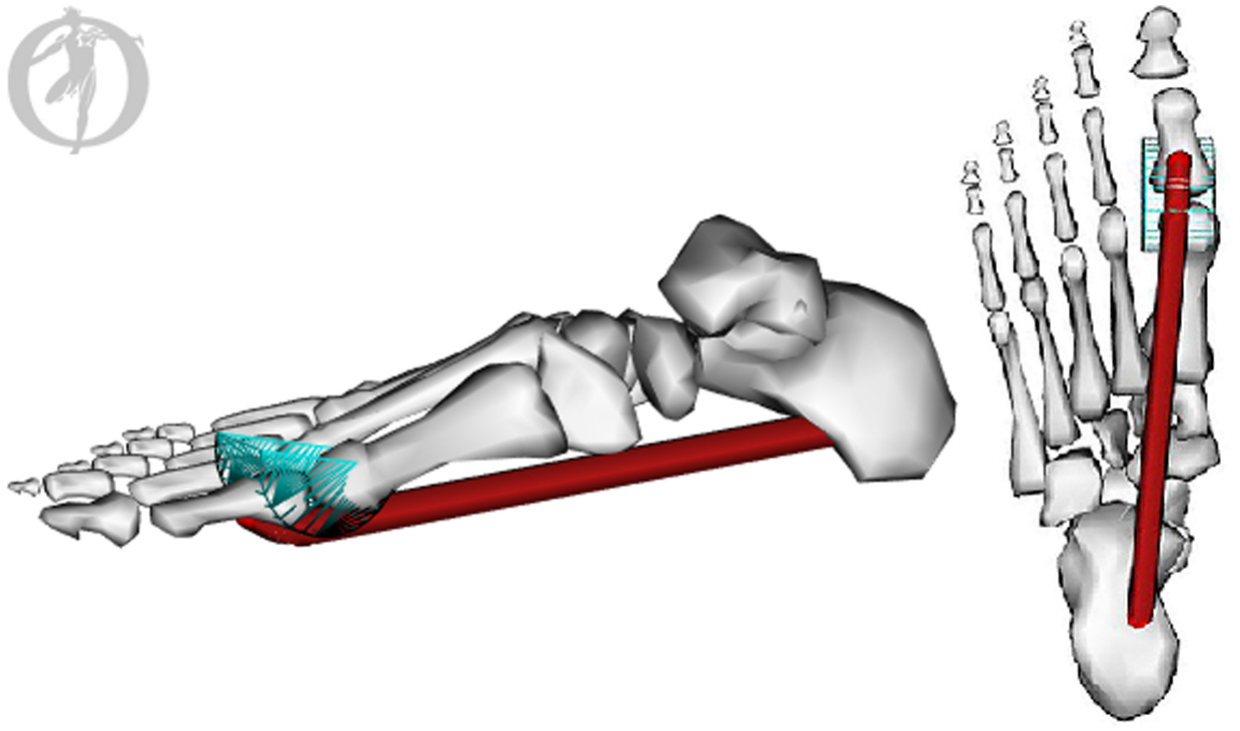
OpenSim Plantar Fascia Model. The tissue displayed represents the first slip of the plantar fascia with an origin on the medial process of the calcaneal tuberosity and an insertion at the base of the proximal phalanx. A wrapping surface was also created around the metatarsophalangeal joint. This image was developed using OpenSim 3.0.1.

A pre-existing SIMM (MusculoGraphics Inc, Evanston, USA) lower body model was modified to include an additional tissue with the anatomical path of the first slip of the PLF (i.e. spanning from the calcaneal tuberosity to the base of the proximal phalanx). Appropriate wrapping surfaces were inserted, before the model was transferred to the open-source software package OpenSim 3.0.1 (Fig 2). Foot length and width were scaled for each subject, however the forefoot and rearfoot segments were not individually scaled. MPJ angle data computed in BodyBuilder (Vicon, Oxford Metrics, Oxford, UK) were imported to allow PLF length changes to be estimated from MPJ angular changes for each subject’s running trials.

Stretch of the PLF due to arch compression was also estimated. Navicular height (measured using 3D motion capture) was expressed continuously relative to an axis defining the base of the foot. This axis was defined from the rearfoot anteroposterior axis with an origin at RMP5a. Because inversion/eversion does not affect the location of the navicular marker in the rearfoot coordinate system, the navicular height measurement was not limited by inversion/eversion as it would be in a two-dimension planar analysis.

The estimated PLF ‘resting length’ from the scaled OpenSim model (when MPJ angle = 0°) along with navicular height data from an unweighted motion capture trial were entered into a simple geometric model. Assuming that the distance from the navicular marker to the end of the arch is constant, we used the variable navicular height data obtained from the running trials to trigonometrically compute subject-specific dynamic PLF length changes due to arch compression across the stance phase of running.

The estimated length changes of the PLF arising from the MPJ angle (OpenSim model) and arch compression (geometric model) were combined to estimate how the PLF changed length between successive time points throughout the stance phase of all conditions. This length change was then normalized to the estimated PLF ‘resting length’ as a measure of functional PLF strain. It should be noted that these length and strain values are unlikely to be the exact strain of the PLF tissue itself since it is difficult to estimate the PLF slack length, but offer a functionally relevant means to standardize PLF dynamics.

### Insert Design

Prior to biomechanical testing, subjects met with a certified podiatrist and were fitted for a pair of orthotic-style inserts (referred to as the ‘insert’) using 3D scans of the subjects feet in a non-weight bearing neutral sub-talar joint position (ScanAny, Orthotech laboratories, Melbourne, Australia). The maximum arch height of the insert replicated the peak height of the MLA in the non-weight-bearing position. The insert was intended to maximally restrict arch compression during weight bearing, within comfort limits of the participant.

The inserts were made from 4mm polypropylene, had a high density arch fill (shore value ~350–400), a four degree intrinsic rearfoot grind, and balanced forefoot intrinsic and maximum arch congruency. The material properties of the insert were tested using an Instron machine (Instron Model 8874, Illinois Tool Works Inc., USA) and Instron Dynamic Software (WaveMatrix 1.2) and it was determined that the inserts return less than 0.4J of energy during running [5].

### Statistical Analysis

All data was screened for normality following which, results were analyzed using a 2 x 3 mixed model (within and between groups) analysis of variance (ANOVA) to determine main effects of condition (barefoot, shod, insert) and foot strike technique (FFS, RFS). When necessary, post hoc testing (Bonferroni) was undertaken to determine where significance was present. This procedure was applied to PLF strain values, MPJ peak angles, peak and average net moments, peak powers and work, onset of active MPJ extension, and timing of peak strain. SPSS 20.0 (Chicago, USA) was used to perform all statistical analyses with a p value limit set to 0.05 for ANOVA analysis, and adjusted to 0.0167 in Bonferroni post hoc analyses.

## Results

All dependent variables were assessed for significant differences between condition (barefoot, shod, insert) and foot strike posture (FFS, RFS). Where no reference is made to a main effect of foot strike posture, no significant differences were found to exist between FFS and RFS runners for that variable. In such cases, a whole-sample (FFS and RFS) average is presented.

### Plantar Fascia Strain—General Observations

**Fig 3:**
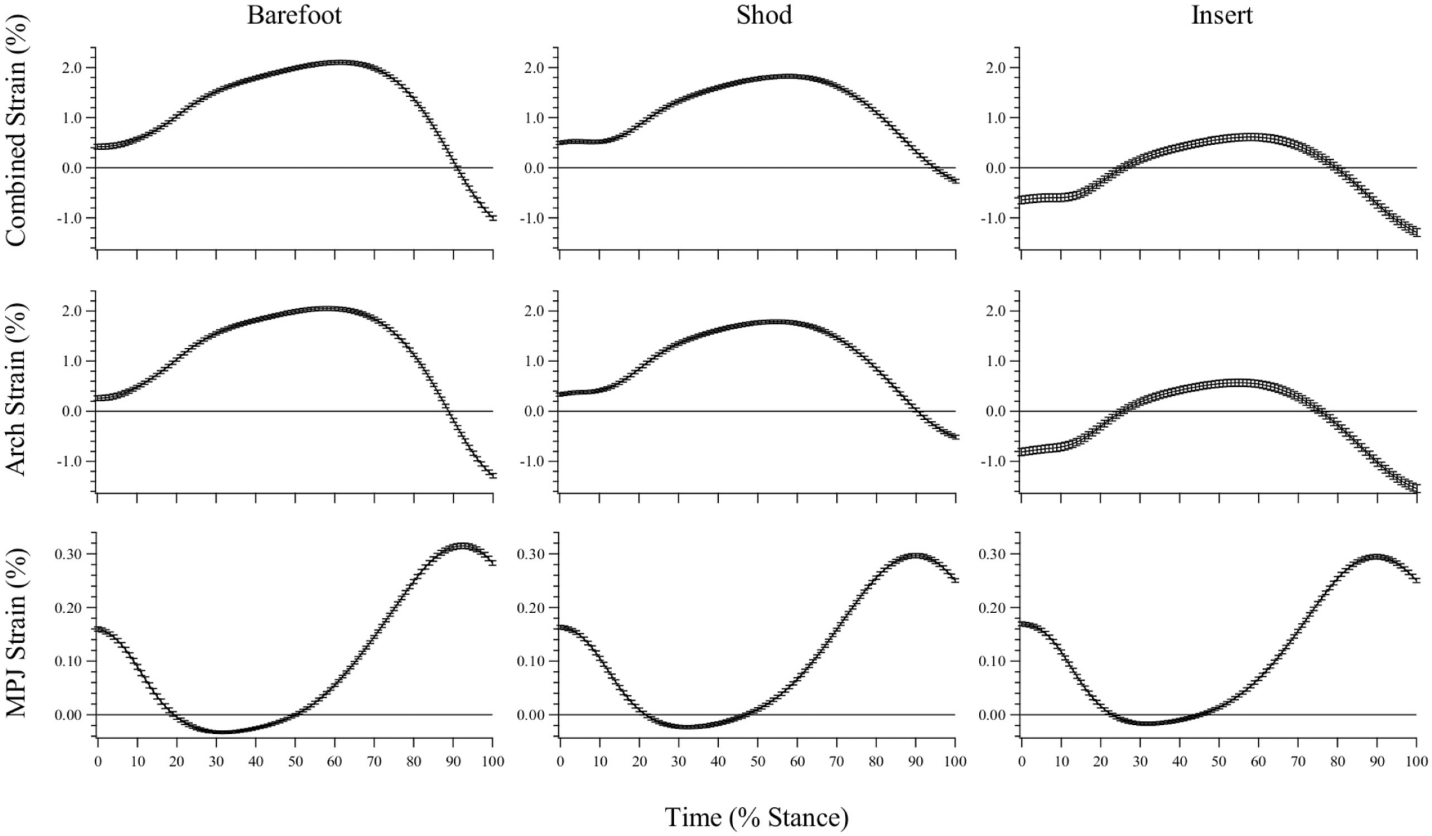
Estimated plantar fascia strain during stance phase. Presented as the combined effect of arch compression and metatarsophalangeal joint (MPJ) angle, the effect of arch compression only, and the effect of MPJ angle only (mean ± sd). Strain values ≤ 0 represent lengths at which the PLF is estimated to be slack.

For each condition (barefoot, shod, insert), three stance phase PLF strain profiles are presented; (1) ‘Combined Strain’: the combined PLF strain generated when the effects of arch compression and MPJ excursion are summed (for each time normalized frame of stance phase), (2) ‘Arch Strain’: the strain generated through arch compression alone, when the influence of MPJ excursion is removed, and (3) ‘MPJ Strain’: the strain generated through MPJ excursion alone, when the influence of arch compression is removed (Fig 3).

**Fig 4:**
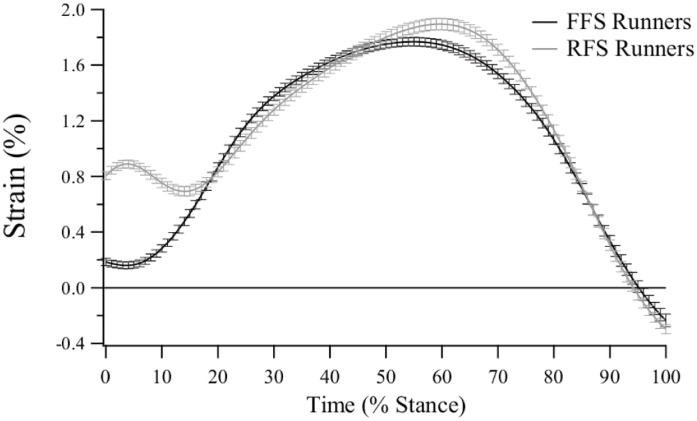
Estimated plantar fascia strain during the stance phase of unrestricted shod running for forefoot strike (FFS) and rearfoot strike (RFS) runners (mean ± sd).

The combined strain curve for unrestricted shod running shows that the average PLF length from all participants (both FFS and RFS) stretches slightly beyond the estimated resting length at foot contact (~0.5% strain; Fig 3). The PLF undergoes a gradual increase in length as the foot is loaded during the stance phase, reaching a peak strain of ~1.8% at ~65% of stance. In FFS running, PLF lengthening initiates shortly after foot strike (~5% of stance), but in RFS running the initiation of lengthening occurs at ~15% of stance, concomitant with the time the ball of the foot makes contact with the treadmill after initial heel strike (Fig 4).

The insert restricted maximum arch compression by approximately 70% when compared to unrestricted shod running and consequentially resulted in lower strain values throughout the entire stance phase. It should be noted that the PLF length only surpasses the estimated resting length between ~25%-80% of the stance phase in the insert condition (Fig 3). The negative strain values should be regarded as a slack PLF length, not as the PLF shortening beyond the resting length.

### Maximum Plantar Fascia Strain

Maximum PLF strain values are presented in Table 1. Maximum strain for the combined effect of arch compression and MPJ flexion was significantly different between the barefoot vs. insert (p < 0.001), and shod vs. insert conditions (p = 0.001), with the barefoot condition resulting in the highest peak strain values, followed by the shod condition, and finally, the insert condition produced the lowest peak strain values. These results were consistent with the maximum PLF strain derived from arch compression only. Maximum strain as a result of MPJ excursion did not differ between the shod and insert conditions (p = 1.000), however the maximum MPJ-derived strain in the barefoot condition was significantly greater than both the shod (p = 0.001) and insert (p = 0.003) conditions (Table 1).

**Table 1:** Maximum plantar fascia (PLF) strain.

	Maximum PLF Strain from Arch Compression and Metatarsophalangeal Joint Angle (%)		Maximum PLF Strain from Arch Compression Only (%)		Maximum PLF Strain from Metatarsophalangeal Joint Angle Only (%)	
**Group**	**Average**	**Standard Deviation**	**Average**	**Standard Deviation**	**Average**	**Standard Deviation**
Barefoot	2.122	0.612	2.068	0.623	0.318	0.074
Shod	1.852	0.581	1.809	0.581	0.299	0.06
Insert	0.636	1.212	0.591	1.195	0.299	0.063
**Statistics**	**Difference**	**p Value**	**Difference**	**p Value**	**Difference**	**p Value**
Barefoot / Shod	0.27	0.059	0.259	0.068	0.019	0.001*
Barefoot / Insert	1.486	<0.001*	1.477	<0.001*	0.019	0.003*
Shod / Insert	1.216	0.001*	1.218	0.001*	0	1

* Significantly different after Bonferroni correction (p < 0.0167). Average values represent combined RFS and FFS data.

As a percentage of stance phase, peak strain occurred significantly earlier in the barefoot condition (62.2±4.4%) when compared to the shod (p < 0.001; 58.4±5.0%) and insert (p < 0.001; 58.8±5.2%) conditions. The latter two conditions were not significantly different from one another (p = 1.000), and no main effect of foot strike posture was present for this variable (p = 0.122). The FFS/RFS shod strain curves are presented in Fig 4.

### Metatarsophalangeal Joint Dynamics—General Observations

**Fig 5:**
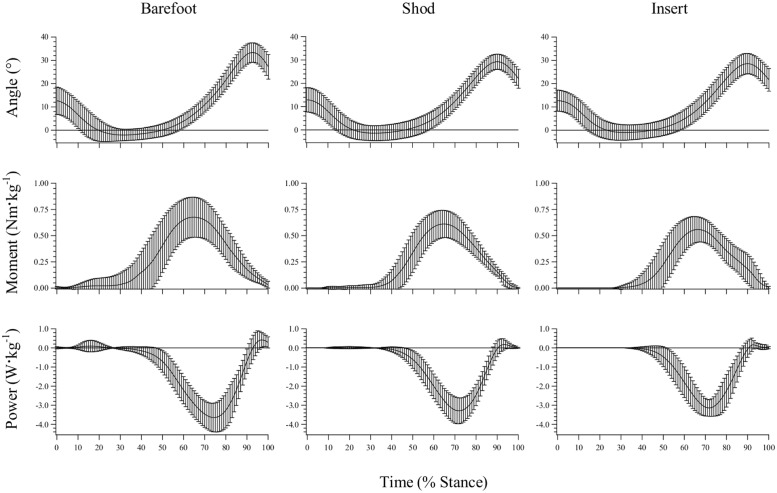
Metatarsophalangeal joint angle, net moment and power traces during stance phase (mean ± sd).

The MPJ underwent extension (plantarflexion) during the first ~35% of stance. This was followed by a period of flexion (dorsiflexion), which ended when the joint began to extend at ~90% of stance (Fig 5). The MPJ experienced minimal loading in the first ~35% of the stance phase due to the GRF center of pressure being positioned posterior to the MPJ center. Thus, the MPJ also exhibited minimal joint power in the first ~35% of stance. In the last two thirds of stance the MPJ was under a net extension moment and, since the joint was flexing with the rising heel, it almost exclusively functioned to absorb energy (negative power) for the remainder of stance phase (Fig 5).

### Metatarsophalangeal Joint Kinematics

**Fig 6:**
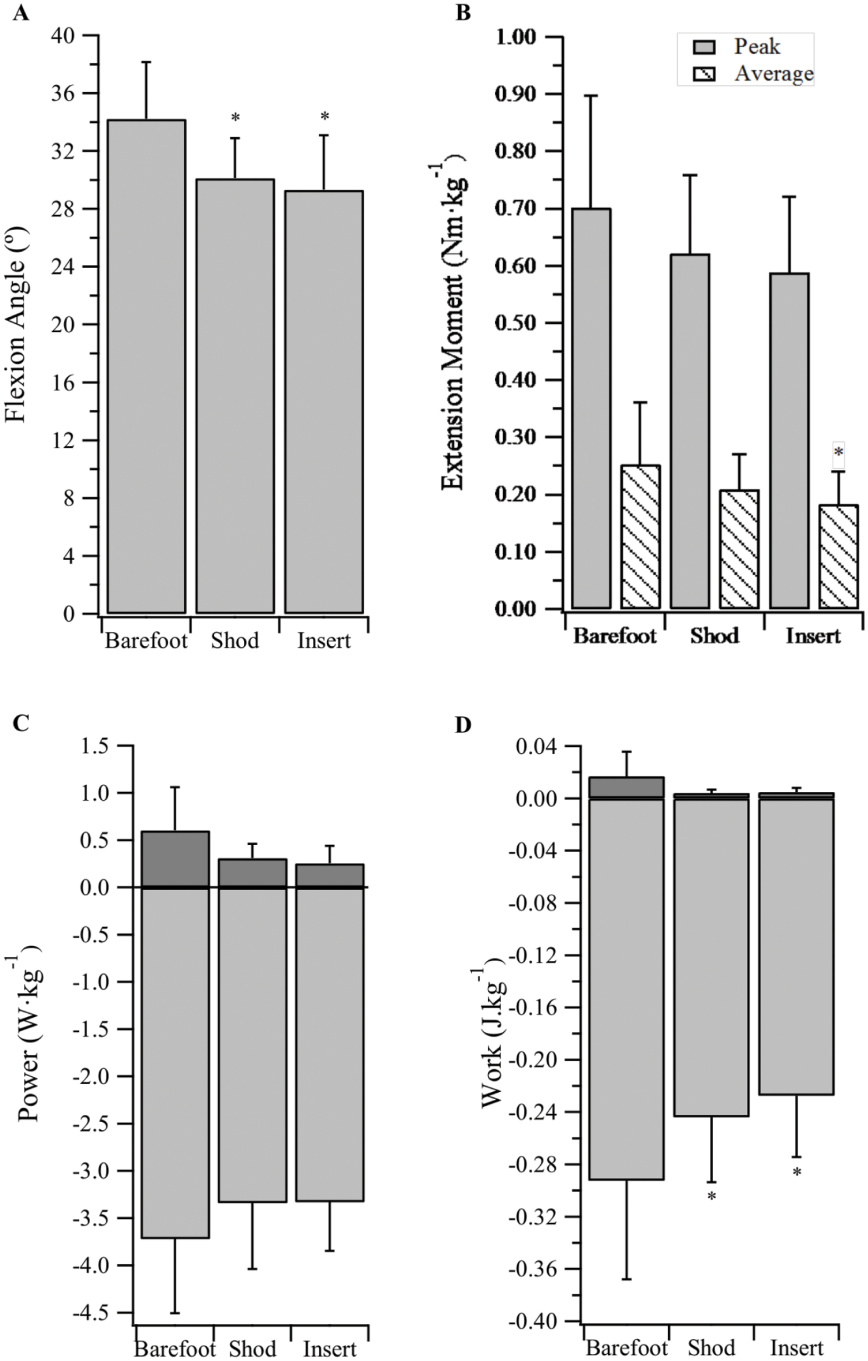
Metatarsophalangeal joint dynamics. (A) Peak flexion angle, (B) peak and average net extension moment, (C) peak positive and negative power, and (D) positive and negative work, during stance phase (mean ± sd). * Significantly different from ‘Barefoot’ condition (p<0.0167). ** Significantly different from ‘Shod’ condition (p<0.0167).

Whole-sample maximum MPJ flexion angles for the barefoot, shod and insert conditions were 34.2±3.9°, 30.1±2.8°, and 29.3±3.8°, respectively (Fig 6A). A main effect of condition was detected. Peak flexion angles in the barefoot condition were significantly greater than the shod (p < 0.001) and insert (p < 0.001) conditions, however the shod and insert conditions were not significantly different (p = 1.000).

The onset of active MPJ extension occurred at 93.7±2.7%, 90.9±2.6% and 91.2±3.4% of stance for the barefoot, shod and insert conditions, respectively. A main effect was observed for condition, with post hoc analyses (Bonferroni) identifying the barefoot onset occurred significantly later than the shod (p < 0.001) and insert (p = 0.005) conditions. The insert did not result in an earlier onset of active extension when compared to the shod condition (p = 1.000).

### Metatarsophalangeal Joint Kinetics

The barefoot condition produced a significantly greater average net extension moment (0.25±0.11Nm·kg^-1^) than the insert condition (0.18±0.06Nm·kg^-1^; p = 0.009; Fig 6B), however no significant differences were present for peak net moment (Fig 6B), peak positive and negative power (Fig 6C), and positive work (Fig 6D). As illustrated in Fig 6D, there was a main effect of condition for negative MPJ work, with subjects absorbing significantly greater magnitudes of power at the MPJ when barefoot, compared to running shod or with the insert (p < 0.0167).

## Discussion

### Plantar Fascia Strain and Elastic Energy Storage

The main purpose of this study was to explore the spring function of the PLF in running by integrating the effects of MLA compression and MPJ excursion. Although the elastic energy storage potential of the PLF has been acknowledged throughout the literature [3, 6, 7], a more detailed analysis of its in vivo functionality during running is lacking. Our findings suggest the PLF conforms to the general stretch-shorten model of elastic structures (i.e. lengthening between foot strike and midstance, and shortening between midstance and toe off). Strain was progressively increased until a peak value was reached at approximately 60% of stance. During this time the PLF stores elastic energy, which is returned in the final 40% of stance causing the bony structure of the MLA to recoil (Fig 3). The recoiling arch functions to produce gravitational potential energy and kinetic energy to aid propulsion of the body’s center of mass.

### Modulation of Plantar Fascia Strain—Shod and Barefoot

During unrestricted shod and barefoot running, the PLF was observed to develop strain via both arch compression and MPJ flexion. However, when we assessed the relative contributions, arch compression presented as the primary strain inducing mechanism, with the MPJ having a minor influence (Fig 3). This can be explained by the small PLF moment arm associated with the MPJ.

Significantly greater MPJ-derived strain was observed in the barefoot condition when compared to shod running; the result of a significantly greater peak MPJ flexion angle. Conversely, the two conditions did not produce significantly different peak arch strain values, likely due to the minimalist nature of the footwear with respect to arch restriction.

**Fig 7:**
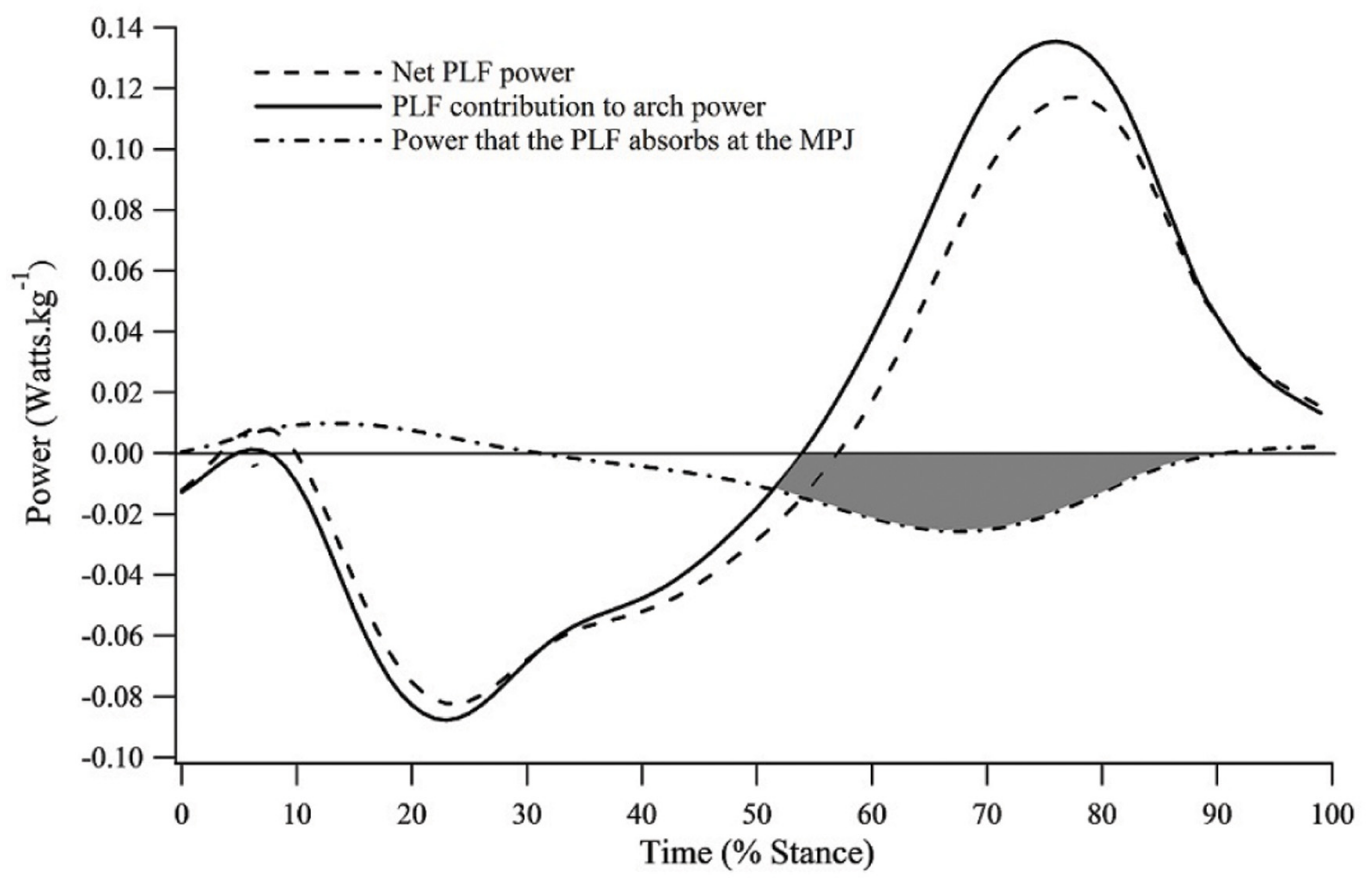
Plantar fascia energy transfer mechanism. Plantar fascia (PLF) net power is represented by the evenly broken line; PLF contribution to arch power is represented by the unbroken line; and power that the PLF absorbs at the metatarsophalangeal joint (MPJ) is represented by the unevenly broken line. The shaded region represents the energy absorbed at the MPJ during the propulsive phase of ground contact.

Integrating the effects of arch compression and MPJ dynamics led to the discovery of a novel PLF function. In the latter half of stance, the MLA is recoiling and generating positive mechanical power, whilst energy is simultaneously absorbed at the MPJ. In unrestricted shod running, we observed MPJ negative work to account for 11.7%±3.2% of total lower limb joint energy absorption during stance phase. Some of this absorbed energy can be transferred by the PLF between the MPJ and recoiling MLA to assist with positive power generation (shaded region, Fig 7). The concept of energy transfer in biological tissue is not a new one. Biarticular lower limb muscles such as rectus femoris and gastrocnemius [29–32] have been found to transfer energy between their proximal and distal joints. However, the PLF has, to our knowledge, not previously been identified as an energy transfer mechanism between energy absorbing and producing structures of the foot.

Fig 7 displays an estimate of PLF power in the shod condition, defined as the product of tissue force and velocity. Force data were obtained using a stiffness value of 187N·mm^-1^ [11, 33] multiplied by PLF length. Velocity was defined as the change in tissue length with respect to the change in time. When length data resulting from arch dynamics is considered independently, the PLF contribution to positive arch power in the second half of stance is substantially greater than the net PLF power derived from the combined PLF length changes (at the arch and MPJ; Fig 7).

The PLF’s energy transfer mechanism has two important benefits; (1) additional energy is transferred between the MPJ and bony MLA which may assist propulsion, and (2) the risk of strain-induced PLF injury is potentially lower because the power the PLF contributes to the arch at push-off does not depend entirely on previously stored elastic strain energy.

### Modulation of Plantar Fascia Strain—Running with Impaired Medial Longitudinal Arch Function

A systematic method of reduced arch compression was employed to assess how the PLF would respond to impaired MLA spring function. When peak compression was restricted by ~70% (compared to unrestricted shod) both the maximum combined strain and that generated by the arch alone were significantly reduced (Fig 3). We predicted the MPJ would begin active extension (plantarflexion) earlier and reach a higher peak flexion (dorsiflexion) angle, providing greater PLF strain and facilitating improved forward propulsion via increased positive work at the joint. Contrary to our hypothesis, maximum strain as a function of MPJ angle was not affected by the insert. There was also no significant difference between the shod and insert conditions for the onset of active MPJ extension, or positive MPJ work (Fig 6D).

The insert caused a ‘slackening’ of the PLF, which subsequently may have prevented the MPJ from achieving the same average net extension moment as in the barefoot condition (Fig 6B). This might occur because the PLF contribution to the MPJ moment is determined by the extent of tissue stretch, which dictates its force. Several muscles also cross the MPJ that can contribute to MPJ moments including the flexor digitorum longus and flexor digitorum brevis. However, these muscles do not appear to compensate for the loss of MPJ moment arising from changes in PLF lengths. As these muscles are thought to play a role in supporting the arch [13, 34–36], we predict that when they are no longer required in this role (i.e. the insert is providing substantial arch support), decreased activation will result in reduced torque generation at the MPJ. This reduction in average net extension moment is likely responsible for the decrease in negative work (energy absorption) observed at the MPJ when the insert condition is compared to the barefoot condition. It is also important to consider that potential benefits of the energy transfer mechanism (described above) may be degraded as a result of this reduced energy absorption.

### Effect of Foot Strike Posture on Plantar Fascia Strain

An additional aim of this study was to identify differences in PLF strain profiles between FFS and RFS runners. The two foot strike postures displayed comparable strain curves throughout 80% of stance phase (Fig 4). For the first 20% of stance however, the two curves are dissimilar, with RFS runners exhibiting significantly larger strain values at initial contact. This finding is consistent with literature describing a link between dorsiflexion at the ankle joint, MPJ flexion and increased PLF strain [37, 38].

Perl, Daoud [19] propose that FFS runners may store more elastic energy in their MLA ligaments throughout stance phase because the arch can be progressively loaded from initial contact, whereas RFS runners can only compress the arch from foot flat. However, it is peak strain that primarily determines resulting elastic energy benefits and, contrary to our hypothesis, this study found no discernible difference between foot strike postures for peak PLF strain. These findings suggest that FFS runners receive no additional performance benefits from the PLF, nor are they at a heightened risk of strain-related PLF injury when compared to RFS runners. No other fundamental dependent variables were affected by foot strike posture.

### Functional Implications

It has previously been suggested that orthotics with high medial profiles can effectively reduce PLF strain [12] and this is supported in the present study. Inserts designed to reduce PLF strain may have functional implications for the treatment of plantar fasciitis given that attenuated PLF strain has been associated with lessened symptoms [39]. From a performance perspective however, the use of rigid arch supporting orthotics has the potential to impair PLF spring function by limiting the maximum strain that can be developed. It may therefore be important that clinicians prescribing orthotics consider the energetic consequences of restricting arch compression [5], especially when the patient partakes in regular endurance running.

Salzler, Bluman [40] assessed running injuries concomitant with the transition from a shod to a barefoot gait. From a sample of ten subjects, eight presented with metatarsal fractures and one with a rupture of the PLF. The increased negative work at the MPJ and the trend for greater peak PLF strain (p = 0.059) observed for the barefoot condition may be linked to these respective injuries, and this should be considered if converting from shod to barefoot running. Endurance athletes however, could potentially benefit from enhanced PLF elastic energy return that may be associated with barefoot running, especially at higher running velocities than used in this study.

### Limitations

We acknowledge that our estimation of PLF strain is used as a means to normalize PLF length changes, and is not necessarily measuring the underlying strain of the tissue relative to a tissue slack length (i.e. the length above which force is first generated in the tissue). Moreover, our strain models were represented by the activity of the hallux and most medial aspect of the MLA, and did not account for curvature of the arch. A true multi-segment foot model (e.g. the Oxford Foot Model [41]) would improve the accuracy of this approach.

Our PLF strain estimate was derived using a resting length obtained from the scaled OpenSim model. The average resting length for all participants was ~170mm, yet Stecco, Corradin [42] report a mean resting length of 120mm. Several factors could have led to such a large discrepancy including the use of cadaver specimens with a mean age of 73 years. These authors also took the measurement from the long axis of the tissue, and although our length estimation shares the same origin, it inserts medial to the long axis. Finally, our participants possessed above-average foot lengths as they were required to be in the shoe size range of US10-12. Nevertheless, we conclude that some level of overestimation of PLF length (resting and dynamic) is likely to have occurred, although this effect is less pronounced because normalized strain values are presented. Ultrasound imaging or radiographic fluoroscopy could potentially be used in future analyses to identify a more accurate resting length of the PLF. These imaging techniques would also allow for greater precision when measuring the PLF moment arm at the MPJ. Recalculating peak PLF strain in the shod condition using a 120mm resting length produced values approximately twofold our reported data (~1.8 to ~3.7% strain), which would subsequently accentuate the PLF’s role as an elastic energy source but would not influence the interpretation of the tissue’s function. The recalculated peak strain of 3.7% does not exceed the realistic range of ligament and tendon tissues.

All running trials were conducted on a force plate instrumented treadmill. It has been documented within the literature that gait kinematics/kinetics can vary between overground and treadmill conditions [43, 44]. Sinclair, Taylor [45] also reported lower PLF strain values when participants ran on a treadmill as opposed to overground. These limitations restrict the interpretation of results to treadmill running, and caution should be taken when applying findings to overground running. Our assessment of MPJ angles, net moments, and powers were limited to the sagittal plane. We acknowledge that a complete analysis of joint dynamics would incorporate transverse and frontal plane movements. Finally, our population sample was limited to young, healthy males with neutral feet. Due to reported differences in PLF properties between genders [11, 46] and age groups [47], these results can only be applied to a similar population to that which was tested.

## Conclusion

We propose that the PLF functions as a valuable elastic spring during running. When the two mechanisms of PLF strain production were dissociated (arch compression and MPJ flexion) a novel energy transfer mechanism was uncovered. PLF strain was primarily dictated by arch compression, however the MPJ was found to absorb energy from impact with the ground, and some portion of this can be transferred between the recoiling MLA, possibly enhancing propulsion. Restricting MLA compression, and hence spring function of the arch, was not compensated for by an increase in MPJ-derived strain. Foot strike posture did not appear to affect any of the fundamental dependent variables measured in this study. These findings not only improve our understanding of the structure-function relationship of the foot, but provide potentially important information for clinical assessments.
